# Supplementary material for: Quality Management System for an IoT Meteorological Sensor Network—Application to Smart Seoul Data of Things (S-DoT)
Source: Sensors (Basel). 2023 Feb 21;23(5):2384. doi: 10.3390/s23052384 (PMC10037411; doi:10.3390/s23052384)
Supplement: Supplementary file 1 [file sensors-23-02384-s001.zip › sensors-2209776-supplementary.pdf]

sensors

MDPI

Supplementary Materials

Quality Management System for an IoT Meteorological Sensor Network – Application to Smart Seoul Data of Things (S-DoT)

Moon-Soo Park [1,*] and Kitae Baek [2]

[1] Department of Climate and Environment, Sejong University, Seoul 05006, Republic of Korea
[2] Climate Change & Environmental Research Center, Sejong University, Seoul 05006, Republic of Korea
* Correspondence: moonsoo@sejong.ac.kr; Tel.: +82-2-6935-2558

Table S1. Mean bias (MB), mean absolute bias (MAB), and root mean square error (RMSE) for different imputation methods at station ID of V02Q1940941 on 24 July 2021.

Method	MB	MAB	RMSE
Random	+0.09	3.39	4.18
mean	+0.35	2.72	3.13
Locf[1]	-0.005	0.08	0.13
Nocb[2]	-0.003	0.07	0.12
Linear interpolation	-0.004	0.04	0.07
Spline interpolation	-0.007	0.06	0.09
Stineman interpolation	-0.004	0.04	0.06
Simple moving average	-0.007	0.08	0.12
Linear moving average	-0.006	0.07	0.10
Exponential moving average	-0.006	0.06	0.09

[1]locf: last observation carried forward, [2]nocb: next observation carried backward.

Table S2. Mean bias (MB), mean absolute bias (MAB), and root mean square error (RMSE) for different imputation methods at a station ID of V02Q1940142 on 24 July 2021.

Method	MB	MAB	RMSE
Random	0.152	2.71	3.36
mean	-0.011	2.15	2.36
locf	0.011	0.06	0.09
nocb	0.011	0.06	0.10
Linear interpolation	0.011	0.04	0.05
Spline interpolation	0.007	0.05	0.06
Stineman interpolation	0.011	0.04	0.05
Simple moving average	0.011	0.06	0.08
Linear moving average	0.011	0.05	0.07
Exponential moving average	0.012	0.05	0.06
Kalman StructTS	0.011	0.04	0.05
Kalman auto.arima	0.009	0.04	0.06
